# Concurrent Daily and Non-Daily Use of Heated Tobacco Products with Combustible Cigarettes: Findings from the 2018 ITC Japan Survey

**DOI:** 10.3390/ijerph17062098

**Published:** 2020-03-22

**Authors:** Edward Sutanto, Connor Miller, Danielle M. Smith, Ron Borland, Andrew Hyland, K. Michael Cummings, Anne C.K. Quah, Steve Shaowei Xu, Geoffrey T. Fong, Janine Ouimet, Itsuro Yoshimi, Yumiko Mochizuki, Takahiro Tabuchi, Richard J. O’Connor, Maciej L. Goniewicz

**Affiliations:** 1 Division of Cancer Prevention and Population Sciences, Department of Health Behaviors, Roswell Park Comprehensive Cancer Center, Buffalo, NY 14263, USA; edward.sutanto@roswellpark.org (E.S.); Connor.Miller@RoswellPark.org (C.M.); Danielle.Smith@RoswellPark.org (D.M.S.); Andrew.Hyland@RoswellPark.org (A.H.);; 2 Melbourne School of Psychological Sciences, The University of Melbourne, Melbourne 3010, Australia; rborland@unimelb.edu.au; 3 Department of Psychiatry & Behavioral Sciences, Medical University of South Carolina, Charleston, SC 29425, USA; cummingk@musc.edu; 4 Department of Psychology, University of Waterloo, Waterloo, ON N2L 3G1, Canada; ackquah@uwaterloo.ca (A.C.K.Q.); s4xu@uwaterloo.ca (S.S.X.); geoffrey.fong@uwaterloo.ca (G.T.F.); j2ouimet@uwaterloo.ca (J.O.); 5 Ontario Institute for Cancer Research, Toronto, ON M5G 0A3, Canada; 6 Division of Tobacco Policy Research, National Cancer Center Japan, Tokyo 104-0045, Japan; iyoshimi@ncc.go.jp; 7 Japan Cancer Society, Tokyo 100-0006, Japan; mochizuki@jcancer.jp; 8 Cancer Control Center, Osaka International Cancer Institute, Osaka 537-8511, Japan; tabuchitak@gmail.com

**Keywords:** concurrent use, dual use, heated tobacco products, heat-not-burn, combustible cigarettes

## Abstract

Use of heated tobacco products (HTPs) among current smokers is becoming increasingly popular in Japan. This study aims to compare characteristics and tobacco-related behaviors among concurrent users of HTPs and combustible cigarettes (*n* = 644) with exclusive smokers (*n* = 3194) or exclusive HTP users (*n* = 164). The secondary aim was to explore heterogeneity within concurrent use subgroups. Data were from Wave 1 of the ITC Japan Survey, a nationally representative web survey conducted from February to March 2018. Concurrent cigarette-HTP users were younger and wealthier than exclusive smokers. However, there were no difference in the frequency of smoking, number of cigarettes per day, and smoking cessation behaviors between the two groups, suggesting that HTPs reinforce nicotine dependence. Compared to exclusive HTP users, concurrent cigarette-HTP users reported higher frequency of non-daily HTP use, and lower number of tobacco-containing inserts per day. Almost all concurrent cigarette-HTP users smoked every day (93.9%); 48.4% both smoked and used HTPs daily (dual daily users, *n* = 396), while 45.5% were daily smokers and non-daily HTP users (predominant smokers, *n* = 213). Concurrent user subgroups differed from each other on age, tobacco use behaviors, and quit intention. Alongside heterogeneity between concurrent and exclusive product users, differences across concurrent use subgroups highlight the importance of considering frequency of use in characterizing poly-tobacco users.

## 1. Introduction

Combustible cigarettes (herein referred to as “cigarettes”) and heated tobacco products (HTPs) are the two most commonly used tobacco products in Japan [1,2,3]. Contemporary HTPs were first introduced in Japan through the launch of IQOS by Philip Morris International in 2014 [1]). Since then, Japan HTP market have rapidly evolved into the most developed HTP market worldwide, accounting for 85% of the global HTP market in 2018 [4]). Due to sale prohibition under the Pharmaceutical Affair Act, nicotine vaping products (NVPs), which have achieved popularity in many countries, are not common in Japan [5]. While a declining trend in smoking prevalence in Japan has been observed in recent years [6], HTP use has grown in popularity [1]. Studies from 2017 and 2018 have estimated about two-thirds of HTP users concurrently smoked cigarettes [1,7]. A higher estimate was reported in South Korea, where 96.2% of current HTP users were also current smokers in 2018 [8]. Yet, while concurrent use of cigarettes and HTPs (herein referred to as “concurrent cigarette-HTP use”) is common, little is known about characteristics and tobacco use behaviors of these users, and how these compare to exclusive smokers or exclusive HTP users. Concurrent use of multiple tobacco products has been reported to be an unstable use pattern [9,10,11,12]. If HTPs were to serve as an effective substitute for cigarettes, concurrent cigarette-HTP use could represent a transitional behavioral state toward smoking cessation. Conversely, if HTPs serve as complementary products, concurrent cigarette-HTP use may contribute to sustained cigarette use [8].

While the harm reduction potential of certain alternative tobacco products (e.g., snus, NVPs) has undergone scientific scrutiny [13,14,15], less is known about the absolute and relative health effects of HTPs, particularly the relative risk of concurrent cigarette-HTP use compared with exclusive smoking. While many concurrent cigarette-NVP users report that they use NVPs to reduce smoking [16], studies have observed similar or higher concentrations of tobacco-related toxicants in biospecimens of concurrent cigarette-NVP users compared to exclusive smokers [17,18]. Even if concurrent users reduce the number of cigarettes smoked per day, it may not result in a meaningful reduction toward one’s smoking-related mortality risk (as opposed to complete smoking abstinence) [19,20,21]. Limited studies have shown HTP emissions contain higher concentrations of toxicants than observed for NVPs [22,23], indicating that HTP use patterns warrant particular scrutiny.

Concurrent use is a term that has been commonly used in literature to describe heterogenous group of users of two or more tobacco products. However, concurrent users differ in a wide-ranging set of tobacco behaviors [24], e.g., frequency and amount of each product used. This concept has previously been evaluated for concurrent cigarette-NVP users from four different countries (the United States (US), England, Australia, and Canada) by Borland et al. [24]. In their study, four subgroups of concurrent cigarette-NVP users were described, differing in nicotine dependence, quit behaviors, and attitudes toward tobacco products [24]. Based on frequency of each product use, the study classified concurrent cigarette-NVP users to: (1) Dual daily users (those who use both cigarette and NVP daily), (2) Predominant smokers (those who use cigarette daily and NVP less than daily), (3) Predominant vapers (those who use NVP daily and cigarette less than daily), and (4) Concurrent non-daily users (those who use both cigarette and NVP less than daily) [24]. In the present study, we attempted to implement analogous classification to the concurrent cigarette-HTP users in Japan.

Using data from the 2018 International Tobacco Control (ITC) Japan Survey, we performed analysis of concurrent cigarette-HTP users addressing two primary aims. The first aim was to characterize and compare concurrent cigarette-HTP users with exclusive smokers and exclusive HTP users according to sociodemographic and tobacco-related characteristics. The second aim was to categorize concurrent cigarette-HTP users into four subgroups based on the frequency of product use and compare four concurrent cigarette-HTP user subgroups to exclusive smokers, exclusive HTP users, and from each other. 

## 2. Materials and Methods 

### 2.1. Data Source

We analyzed data from the ITC Japan Survey Wave 1, a web-based survey administered by Rakuten Insight and conducted from February to March 2018. The sampling frame of the survey was an existing Rakuten Insight panel that was nationally representative of Japanese cigarette smokers, HTP users, and non-users. Further quotas based on region of residence, gender, and age were applied to ensure final sample was proportional to stratum sizes based on Japan census data. Adult residents of Japan (aged 20 and older [the legal age to purchase tobacco], *n* = 4615) were sampled as participants of the survey. Participants completed an online survey, consisting of questions on cigarette and HTP use, and demographic measures, after eligibility screening. Ethical approval for this study was obtained from the Office of Research Ethics University of Waterloo (ORE#31428).

### 2.2. Measures

#### 2.2.1. User Definitions 

In this study, we defined *current HTP users* (*n* = 808) as participants who used HTPs at least once a month at the time of survey, and *current smokers* (*n* = 3838) as participants who smoked cigarettes at least once a month. *Exclusive users* of HTPs (*n* = 164) or cigarettes (*n* = 3194) were defined as participants who only used one of the two products at least once a month, while *concurrent cigarette-HTP users* (*n* = 644) reported monthly use of both HTPs and cigarettes. Detailed questions to ascertain participant status to each tobacco product use category have been described elsewhere [7].

In line with the typology proposed by Borland et al. [24], we categorized concurrent cigarette-HTP users into four subgroups based on the frequency of tobacco product use (daily vs non-daily use). This resulted in the following categories: (1) *Dual daily users* (those who used both cigarettes and HTPs daily, *n* = 396); (2) *Predominant smokers* (those who use cigarettes daily and HTPs non-daily, *n* = 213); (3) *Predominant HTP users* (those who used HTPs daily and cigarettes non-daily, *n* = 4); and (4) *Concurrent non-daily users* (those who used both cigarettes and HTPs non-daily, *n* = 31). 

#### 2.2.2. Sociodemographic Measures

Age (in years) was categorized into 20–29, 30–39, 40–59, or 60 and older. Gender was categorized into male or female. Income was categorized into low (4,000,000 Japanese Yen or less), moderate (4,000,001-6,000,000 Japanese Yen), high (more than 6,000,000 Japanese Yen), or refused/do not know. Education was categorized into low (junior high school/vocational school/high school), moderate (junior college/technical college), high (undergraduate/postgraduate), or other/refused/do not know.

#### 2.2.3. Pattern of Product Use 

Frequencies of smoking and HTP use were categorized into two groups: daily and non-daily. Non-daily user group included those who reported weekly or monthly use of the product. Two measures from the Fagerstrom Test for Nicotine Dependence (FTND) were adapted for this study [25]. First, for exclusive smokers and exclusive HTP users, time to first tobacco product use was defined as time to first cigarette and time to first HTP use, respectively. Since concurrent cigarette-HTP users reported time to first both products independently, we used the shorter time as time to first tobacco product use. Second, we included the number of cigarettes smoked per day (CPD) and tobacco-containing inserts per day. The number of tobacco-containing inserts per day was only assessed for daily and weekly HTP users as the survey did not ask the number of tobacco-containing inserts per day to monthly HTP users. While both measures have been validated for nicotine dependence in cigarette smokers [25], it has not been validated yet for HTP users. 

#### 2.2.4. Beliefs toward HTPs and Cigarettes

Beliefs toward HTPs and cigarettes were assessed only among participants who were aware of HTPs, which are (1) exclusive HTP users (*n* = 164), (2) concurrent cigarette-HTP users (*n* = 644), and (3) fraction of exclusive smokers (*n* = 2970) who answered ‘yes’ to the question ‘Have you ever heard about electronic “heat-not-burn” products that heat tobacco instead of burning it? These products use battery power to heat capsule, pods, or cigarette-like sticks that contain tobacco. These include products such as IQOS, Ploom TECH, and glo’.

We assessed participants’ perceived harm of HTPs to user with the following question: ‘Compared to smoking cigarettes, how harmful do you think using a heat-not-burn tobacco product is?’. We also assessed participants’ perceived harm of HTP secondhand emissions with the following question: ‘Compared to smoking cigarettes, how harmful do you think the emissions from heat-not-burn tobacco product are to other people?’. In addition to ‘refused’ (recoded as missing) or ‘do not know’, five-point scales were used (from ‘much more harmful’ to ‘much less harmful’) as options. Participants’ perceived addictiveness of HTPs compared to cigarettes was assessed using the following question: ‘Compared to smoking cigarettes, do you think using heat-not-burn products is…?’. In addition to ‘refused’ (recoded as missing) or ‘do not know’, five-point scales were used (from ‘much more addictive’ to ‘much less addictive’) as options.

We examined participants’ views on social norms using the following two questions: ‘What do you think the general public’s attitude is towards smoking cigarettes?’ and ‘What do you think the general public’s attitude is towards using heat-not-burn products?’. In addition to ‘refused’ (recoded as missing) or ‘do not know’, five-point scales were used (from ‘strongly approves’ to ‘strongly disapproves’) as options. Participants’ overall attitudes to cigarettes were assessed using the following question: ‘What is your overall opinion of smoking cigarettes?’. Participants who were aware of HTPs had their overall attitudes to HTPs assessed similarly. In addition to ‘refused’ (recoded as missing) or ‘do not know’, five-point scales were used (from ‘very positive’ to ‘very negative’) as options.

#### 2.2.5. Smoking Cessation-Related Behaviors

Past smoking cessation attempts were examined using the following question: ‘How many quit attempts have you made in the last 12 months? If none, enter zero.’ Those whose answer corresponded with at least one attempt were recoded to have made a smoking cessation attempt in the previous year. Quit intention was assessed using the following questions: ‘Are you planning to quit smoking cigarettes…’. The following options were given ‘within the next month’, ‘between 1-6 months from now’, ‘sometime in the future, beyond 6 months’, ‘not planning to quit’, ‘refused’, and ‘do not know’. Those who responded ‘within the next month’ and ‘between 1-6 months from now’ were classified as planning to quit smoking cigarettes in the next 6 months. 

### 2.3. Statistical Analysis

We presented descriptive statistics of the study population in weighted percentages and 95% confidence intervals [95% CI] for categorical variables and median with interquartile ranges [IQR] for continuous variables. The primary analysis consisted of cross-tabulation using Rao-Scott Chi-square tests. Normality for CPD and the number of tobacco-containing inserts per day was tested with the Shapiro-Francia test. Due to the non-normal distribution for these variables, Wilcoxon-Mann-Whitney was employed to examine the difference between two groups and Kruskal-Wallis with post-hoc Dunn’s test examined the difference between more than two groups. 

We began by comparing concurrent cigarette-HTP users to exclusive smokers and exclusive HTP users. Based on previous studies that established daily users differed from non-daily users on wide range of measures [24,26], we compared concurrent daily users (comprised of dual daily users and predominant smokers) with concurrent non-daily users. We then compared concurrent daily users to exclusive daily smokers and exclusive daily HTP users, along with concurrent non-daily users to exclusive non-daily smokers and exclusive non-daily HTP users. Statistical analyses were performed using *svy* commands in Stata SE version 14.2 (StataCorp, College Station, TX, USA). All tests were two-tailed and considered significant at *p* < 0.05; however, due to multiple comparisons we made in this study, we are cautious in interpreting differences with *p >* 0.001. Further details on the weighting and sampling strategies are provided in the ITC Japan Survey Technical Report (https://itcproject.s3.amazonaws.com/uploads/documents/JP1-1.5_Technical_Report_March_102020_Final.pdf).

## 3. Results

### 3.1. Proportion and Characteristics of Exclusive and Concurrent User of Cigarette and HTP

#### 3.1.1. Proportion of Exclusive and Concurrent User of Cigarette and HTP

**Figure 1:**
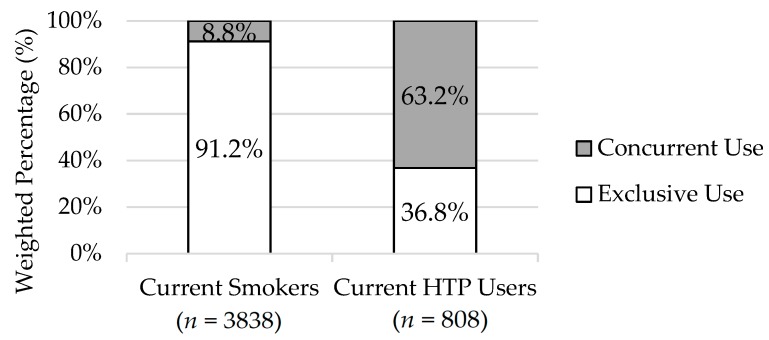
The proportion of exclusive and concurrent use among current smokers (exclusive smokers + concurrent cigarette-HTP users) and current HTPs users (exclusive HTP users + concurrent cigarette-HTP users) in Japan in 2018. The values represented weighted percentages.

Figure 1 shows the proportion of exclusive and concurrent use among current smokers and current HTP users in Japan in 2018. While concurrent cigarette-HTP users only constituted around one-tenth of current smokers (8.8% [8.0–9.7%]), they constituted a majority of current HTP users (63.2% [58.3–67.9%]).

#### 3.1.2. Sociodemographic Characteristics of Exclusive and Concurrent User of Cigarette and HTP

General characteristics of exclusive smokers, concurrent cigarette-HTP users, and exclusive HTP users are shown in Table 1. Compared to exclusive smokers, a greater proportion of concurrent cigarette-HTP users were male, younger, have higher household income, and higher education. Compared to concurrent cigarette-HTP users, a greater proportion of exclusive HTP users belongs to age group of 40–59.

#### 3.1.3. Pattern of Product Use of Exclusive and Concurrent User of Cigarette and HTP

No significant difference was observed in the frequency of smoking between exclusive smokers and concurrent cigarette-HTP users, nor CPD between both groups (15.0 [10.0–20.0] vs. 15.0 [10.0–20.0]). Frequency of HTP use and the number of tobacco-containing inserts per day were significantly different between concurrent cigarette-HTP users and exclusive HTP users, with concurrent cigarette-HTP users reporting a higher frequency of non-daily HTP use and a lower number of tobacco-containing inserts per day (5.0 [1.4–12.0] vs. 10.0 [5.0–20.0]). About two thirds of concurrent cigarette-HTP users used a tobacco product within 30 min after waking up. Among all concurrent cigarette-HTP users, 35.7% reported smoking cigarettes and 9.1% reported using HTPs as the first tobacco product they use after waking up, while 55.2% reported smoking cigarettes and using HTPs in the same time frame after waking up. 

#### 3.1.4. Beliefs and Smoking Cessation-Related Behaviors of Exclusive and Concurrent User of Cigarette and HTP

Compared to exclusive smokers, a higher proportion of concurrent cigarette-HTP users believe that HTPs are less addictive (20.0% [18.5–21.7%] vs. 42.7% [37.9–47.6%]), less harmful to users (43.7% [41.8–45.7%] vs. 69.7% [64.9–74.0%]), and have less harmful secondhand emissions than cigarettes (50.3% [48.3–52.2%] vs. 71.9% [67.1–76.2%]). Compared to concurrent cigarette-HTP users, a higher proportion of exclusive HTP users believe that HTPs are less harmful to users (69.7% [64.9–74.0%] vs. 88.2% [81.2% vs. 92.8%]) and have less harmful secondhand emissions than cigarettes (71.9% [67.1–76.2%] vs. 86.3% [79.5–91.1%]). Compared to exclusive smokers, a greater proportion of concurrent cigarette-HTP users also reported having positive overall opinions of HTPs (28.0% [26.2–29.8%] vs. 56.2% [51.2–61.1%]) and cigarettes (37.5% [35.6–39.3%] vs. 45.9% [41.0–50.9%]). On smoking cessation-related behaviors, no significant difference was observed between concurrent cigarette-HTP users and exclusive smokers.

### 3.2. Proportion and Characteristics of Subgroups of Concurrent Cigarette-HTP Users

#### 3.2.1. Proportion of Subgroups of Concurrent Cigarette-HTP Users

Table 2 presents concurrent cigarette-HTP users categorized according to frequency of product use. Almost all concurrent cigarette-HTP users were daily smokers (93.9% [91.2–95.9%]). The largest subgroups of concurrent cigarette-HTP users were dual daily users (48.4% [43.5–53.3%]), followed by predominant smokers (45.5% [40.5–50.7%]), concurrent non-daily users (5.6% [3.7–8.3%]), and predominant HTP users (0.5% [0.2–1.3%]).

#### 3.2.2. Sociodemographic Characteristics of Subgroups of Concurrent Cigarette-HTP Users

Characteristics of concurrent daily users and concurrent non-daily users are shown in Table 3. Due to the small number of predominant HTP users (*n* = 4), this subgroup was omitted from concurrent daily user group. The majority of concurrent daily users and concurrent non-daily users were male, have high annual household income, and fell into the high education category (i.e., undergraduate or postgraduate degree). Concurrent non-daily users were younger than concurrent daily users. 

#### 3.2.3. Pattern of Product Use of Subgroups of Concurrent Cigarette-HTP Users

CPD differed between concurrent daily users and concurrent non-daily users; concurrent non-daily users reported lower CPD than concurrent daily users. This is also the case for numbers of tobacco-containing inserts per day. Concurrent non-daily users commonly reported more than 60 min as time to first tobacco product use, while concurrent daily users commonly reported 6 to 30 min. 

#### 3.2.4. Beliefs and Smoking Cessation-Related Behaviors of Subgroups of Concurrent Cigarette-HTP Users

No difference was observed in measures related to belief towards cigarettes and HTPs among concurrent daily users and concurrent non-daily users. Past quit attempts and quit intentions did differ between the two groups; concurrent non-daily users reported at least one quit attempt in the previous 12 months and planned to quit smoking in the forthcoming 6 months in a higher proportion than concurrent daily users. 

### 3.3. Differences among Daily Users

#### 3.3.1. Sociodemographic Characteristics of Daily Users

Table 4 contrasts characteristics of four groups of daily users: predominant smoker, dual daily user, exclusive daily smoker, and exclusive daily HTP user. Compared to exclusive daily smokers, a higher proportion of predominant smokers were male (68.7% vs. 79.5%). Dual daily users appeared to be younger than predominant smokers. Compared to exclusive daily smokers, predominant smokers reported higher annual household incomes. 

#### 3.3.2. Pattern of Product Use of Daily Users

While there was no difference in CPD between exclusive daily smokers and dual daily users (15.0 [10.0–20.0] vs. 15.0 [10.0–20.0]), predominant smokers have higher CPD than exclusive daily smokers (18.0 [10.0–20.0] vs. 15.0 [10.0–20.0]). The number of tobacco-containing inserts per day for predominant smokers was lower than exclusive HTP users (0.7 [0.3–1.4] vs. 10.0 [7.0–20.0]), similarly dual daily users reported lower number of tobacco-containing inserts per day than exclusive HTP users (10.0 [5.0–15.0] vs. 10.0 [7.0–20.0]). Compared to exclusive daily HTP user, dual daily user reported a shorter time to first tobacco product use. 

#### 3.3.3. Beliefs and Smoking Cessation-Related Behaviors of Daily Users

Exclusive daily smokers had the least positive harm and addiction perception of HTPs, while exclusive daily HTP users had the most positive perception of HTPs and concurrent cigarette-HTP users (predominant smokers and dual daily users) in between. No pattern on social norms was noted. There was a gradation on positive overall opinion towards HTPs across the four daily-use categories, the lowest among exclusive daily smokers and the highest among exclusive daily HTP users (28.2% vs 64.7%). Positive overall opinion on cigarettes was mixed; predominant smokers reported the highest proportion of positive opinion compared to exclusive daily smokers (49.1% vs. 37.9%). While there was a difference in future smoking cessation attempts between exclusive daily smokers and dual daily users (8.0% vs. 14.0%), no other smoking cessation behaviors were found to differ significantly 

### 3.4. Differences among Non-Daily Users 

#### 3.4.1. Sociodemographic Characteristics of Subgroups of Non-Daily Users

Comparison of three groups of non-daily users were presented in Table 5. No sociodemographic differences were noted between concurrent non-daily users and exclusive non-daily smokers or exclusive non-daily HTP users. 

#### 3.4.2. Pattern of Product Use of Subgroups of Non-Daily Users

While there was a difference in CPD between exclusive non-daily smokers and concurrent non-daily users (1.4 [0.7–2.9] vs. 2.9 [1.3–6.0]), no difference was observed in the number of tobacco-containing inserts per day between concurrent non-daily users and exclusive non-daily HTP users (1.4 [0.4–2.8] vs. 1.7 [0.7–7.1]). Compared to exclusive non-daily smokers, concurrent non-daily users reported a shorter time to first tobacco product use. 

#### 3.4.3. Beliefs and Smoking Cessation-Related Behaviors of Subgroups of Non-Daily Users

No consistent significant gradation of perceived harm and addictiveness of HTPs relative to cigarettes were noted among three groups of non-daily users. Similarly, no significant and consistent pattern was observed on social norms and positive opinion of using HTPs or smoking cigarettes among three groups of non-daily users. A higher proportion of concurrent non-daily users reported plan to quit smoking cigarettes in the next 6 months compared to exclusive non-daily users (50.6% vs. 25.9%).

## 4. Discussion

### 4.1. Characteristics of Concurrent Cigarette-HTP Users 

Our study found that concurrent cigarette-HTP users differed from exclusive smokers and exclusive HTP users on a number of measures. Concurrent cigarette-HTP users were younger and wealthier than exclusive smokers, indicating important sociodemographic differences between the two tobacco user groups. Notably, this contrasts the findings of an ‘actual use’ study conducted by Philip Morris International in the US, which suggested that uptake of IQOS was more likely among middle-aged smokers than young adults [27]. Prior studies has depicted alternative tobacco products as being popular among young adults, in part due to perceptions of lower risk and marketing strategies targeting younger users [28,29,30]. This is also consistent with the findings of our study as concurrent cigarette-HTP users were younger and perceived HTPs as less harmful than cigarettes. It is unsurprising that the sleek and high tech appearance of HTPs might appeal to the young adult population in Japan [31,32]. The popularity of HTPs among affluent population mirrors the price difference between the two products. The price for a pack of cigarettes and a pack of tobacco-containing inserts for HTPs are comparable (In 2018, the price of a pack of cigarettes was approximately ¥500, similarly, the price of a pack of tobacco containing-inserts for IQOS was approximately ¥500) [7]. However, HTP users need to buy the HTP device which cost ranging from 6 to 18 times the price of a pack of cigarettes [7]. 

We found no differences in CPD between concurrent cigarette-HTP users and exclusive smokers. This suggests that HTPs may have little impact on the consumption of tobacco cigarettes among smokers and may not be seen as a complete substitute product for cigarettes, but rather as a complimentary product. This is consistent with prior research in the Republic of Korea [8]. This implies an increase in total nicotine intake among concurrent cigarette-HTP users. While concurrent cigarette-HTP users consumed a smaller number of tobacco-containing inserts per day than exclusive HTP users, in sum, concurrent cigarette-HTP users could hypothetically have higher exposure to tobacco-related health risks compared to exclusive smokers or exclusive HTP users [33,34]. Additional studies investigating related biomarkers among concurrent cigarette-HTP users are required to confirm this.

Our study found that only about one-tenth of concurrent cigarette-HTP users planned to quit smoking in the next 6 months, which is no different to the proportion among exclusive smokers. This is different from what Borland et al. found for vaping that half of the concurrent cigarette-NVP users planned to quit smoking in the next 6 months compared to one-third of exclusive smokers [24]. Additionally, no differences in quit attempt in the last 12 months were observed between concurrent cigarette-HTP users and exclusive smokers. Those findings highlight an important concern regarding utility of HTPs for smoking cessation in real-world settings. On a more positive note, we found that dual daily cigarette-HTP users were more likely to have plans to quit smoking cigarettes in the next 6 months than exclusive daily smokers. Overall, the levels of interest in quitting appear to be much lower in Japan than in countries studied by Borland et al. [24]. This may be a function of Japanese smokers being more likely to be interested in HTPs for reasons other than as quitting tools, and only begin to think about quitting once they find HTPs to be satisfactory for daily use.

### 4.2. Characteristics of Subgroups of Concurrent Cigarette-HTP Users

Dual daily users were the largest subgroup of concurrent cigarette-HTP users in Japan in 2018. This pattern of concurrent use is different than previously reported among concurrent cigarette-NVP users since a majority of the latter group were predominant smokers [24]. Studies with smokeless tobacco products in the US and Norway have reported that daily use of cigarettes and less than daily/occasional use of smokeless tobacco products was the most common concurrent use pattern observed [35,36]. We hypothesized this difference may arise due country-level differences. Japan has stood out from the rest of developed countries due to its lax national tobacco control policies [37,38]. This would suggest less motivation to quit smoking based on health concerns among its population and likely contributed to the observed variation of pattern of products use. Moreover, HTPs’ unique marketing strategy [39,40] and the similarity between HTPs and cigarettes [32] might be contributing factors to this difference. The products similarity makes it easier for users to switch between two products, and if HTPs deliver a certain better experience (such as cleaner and less smell) [41], then switching can occur for reasons other than health concerns.

Among four daily user groups, with increased frequency in HTP use and reduced frequency of smoking, we found lower degree of harm and addiction perception of HTPs. This finding is unsurprising as the inverse relationship between nicotine products’ harm perceptions and its use have been consistently reported [42,43,44,45]. The differences between three non-daily user groups in this study, however, was less clear. While non-daily smokers have been reported to be a heterogeneous group [26], the lack of consistent difference among non-daily users may be due to some degree of homogeneity within these groups or the small sample size. It remains to be seen whether non-daily users would transition to daily users or non-users, and what are the utilities of HTPs among non-daily users (whether as prevention tool from daily smoking or cessation tool for smokers). 

Taken together, we found that classifying concurrent users in terms of daily versus non-daily use provides a useful metric. A recent study from the US using longitudinal data also showed differential associations between frequency of concurrent cigarette-NVP use with smoking abstinence [46]. Future studies could integrate similar typology to look at longitudinal outcomes that are of vital interest to public health researchers and policymakers. 

### 4.3. Study Limitations

There are several limitations of this study. First, due to the cross-sectional nature of this study, we are unable to precisely determine whether concurrent cigarette-HTP users started as non-users, exclusive smokers, or exclusive HTP users, nor whether they maintain or revert to different tobacco use patterns over time. As such, longitudinal studies are needed to evaluate tobacco use patterns of concurrent cigarette-HTP users over time. Second, it is important to note that Japan’s unique regulatory environment, which only recently adopted a national smoke-free law [47], has had IQOS (a leading HTP brand) introduced much earlier than other countries [5], and placed strong cultural values on cleanliness and respect for others [32,48], could broadly influence the use of HTPs, limiting the potential to generalize to other countries. Third, we were not able to evaluate whether concurrent cigarette-HTP users in this study have also concurrently use other alternative tobacco products, such as NVPs or smokeless tobacco products. Lastly, due to the small sample size, we were unable to conduct a detailed analysis for predominant HTP users. It is unclear whether this is genuinely due to very few of such people or undersampling of this group.

## 5. Conclusions

In 2018, most HTP users in Japan were concurrently smoking cigarettes and majority of concurrent cigarette-HTP users were using both products every day. HTPs appear to reinforce nicotine dependence, rather than serve as smoking cessation tools. The findings from our study question utility of HTPs as substitute products for cigarettes. Different sociodemographic characteristics were observed between concurrent cigarette-HTP users and exclusive smokers. Dual daily users differed in several measures, signifying the importance to distinguish this subgroup from other concurrent cigarette-HTP user subgroups. 

## Figures and Tables

**Table 1:** General characteristics of the study population.

		(A) Exclusive Smokers (*n* = 3194)	(B) Concurrent Cigarette-HTP Users (*n* = 644)	(C) Exclusive HTP Users (*n* = 164)	Significance *
		Weighted % [95% Confidence Interval]			
Sociodemographic					
Gender	Male	69.2 [67.3–71.1]	78.8 [73.8–83.0]	71.8 [63.7–78.6]	A–B: 0.0007
	Female	30.8 [28.9–32.7]	21.2 [17.0–26.2]	28.2 [21.4–36.3]	B–C: NS
Age (years old)	20–29	9.4 [8.3–10.6]	19.7 [15.6–24.5]	12.3 [6.8–21.1]	A–B < 0.0001
	30–39	19.2 [17.7–20.7]	28.3 [24.2–32.8]	23.2 [16.9–31.1]	B–C: 0.0022
	40–59	41.2 [39.4–43.1]	36.4 [31.9–41.1]	57.0 [47.9–65.7]	
	60 and older	30.2 [28.5–32.0]	15.7 [12.2–19.9]	7.4 [3.8–14.2]	
Annual Household Income	Low	28.9 [27.2–30.7]	16.4 [13.2–20.2]	14.1 [9.2–21.0]	A–B < 0.0001
	Moderate	21.9 [20.4–23.5]	24.0 [20.0–28.4]	20.0 [13.9–27.9]	B–C: NS
	High	35.4 [33.7–37.3]	47.9 [43.0–52.8]	60.0 [51.1–68.2]	
	Refused/Do not know	13.7 [12.5–15.1]	11.8 [8.1–16.7]	5.9 [3.0–11.1]	
Education	Low	32.3 [30.6–34.1]	25.7 [22.0–29.8]	24.5 [18.3–32.1]	A–B: 0.0052
	Moderate	22.0 [20.3–23.8]	21.1 [16.6–26.4]	24.2 [18.0–31.7]	B–C: NS
	High	44.4 [42.5–46.3]	52.8 [47.8–57.8]	50.9 [42.0–59.7]	
	Refused/Do not know	1.2 [0.9–1.8]	0.4 [0.1–1.1]	0.4 [0.1–2.8]	
Pattern of Product Use					
Frequency of Smoking	Daily	94.8 [93.9–95.5]	93.9 [91.2–95.9]	NA	A–B: NS
	Non-daily	5.2 [4.5–6.1]	6.1 [4.1–8.8]	NA	
Frequency of HTP Use	Daily	NA	48.8 [43.9–53.8]	89.1 [81.8–93.7]	B–C < 0.0001
	Non-daily	NA	51.1 [46.2–56.0]	10.9 [6.3–18.2]	
Cigarettes per day †		15.0 [10.0–20.0]	15.0 [10.0–20.0]	NA	A–B: NS
Tobacco-containing inserts per day †		NA	5.0 [1.4–12.0]	10.0 [5.0–20.0]	B–C < 0.0001
Time to first tobacco product use	5 min or less	26.4 [24.7–28.1]	26.9 [23.0–31.2]	19.3 [13.4–27.1]	A–B: 0.0384
	6–30 min	39.5 [37.7–41.4]	44.2 [39.2–49.3]	39.0 [30.7–48.1]	B–C: 0.0336
	31–60 min	15.7 [14.3–17.1]	16.6 [13.4–20.5]	19.8 [13.4–28.4]	
	More than 60 min	18.4 [17.0–19.9]	12.3 [9.5–15.7]	21.8 [15.1–30.4]	
Beliefs toward HTPs and cigarettes					
Believes HTPs are much or somewhat less addictive than cigarettes ‡		20.0 [18.5–21.7]	42.7 [37.9–47.6]	47.9 [39.1–56.9]	A–B < 0.0001
					B–C: NS
Believes HTPs are much or somewhat less harmful to users than cigarettes ‡		43.7 [41.8–45.7]	69.7 [64.9–74.0]	88.2 [81.2–92.8]	A–B < 0.0001
					B–C: 0.0001
Believes secondhand emissions from HTPs are much or somewhat less harmful than secondhand emissions from cigarettes ‡		50.3 [48.3–52.2]	71.9 [67.1–76.2]	86.3 [79.5–91.1]	A–B < 0.0001
					B–C < 0.0001
Agrees society strongly or somewhat disapproves using HTPs ‡		23.5 [21.8–25.2]	23.0 [18.5–28.3]	29.5 [22.1–38.2]	A–B < 0.0001
					B–C: NS
Agrees society strongly or somewhat disapproves using cigarettes ‡		64.9 [63.0–66.7]	64.0 [59.4–68.5]	59.8 [50.8–68.2]	A–B: NS
					B–C: NS
Has very positive or positive overall opinions of HTPs ‡		28.0 [26.2–29.8]	56.2 [51.2–61.1]	62.5 [53.3–70.9]	A–B < 0.0001
					B–C: NS
Has very positive or positive overall opinions of cigarettes ‡		37.5 [35.6–39.3]	45.9 [41.0–50.9]	34.4 [26.6–43.2]	A–B: 0.0075
					B–C: 0.0119
Smoking Cessation-related Behaviors					
Attempted to quit at least once in the last 12 months		50.4 [47.9–52.9]	54.2 [47.4–60.9]	NA	A–B: NS
Plans to quit smoking cigarettes in the next 6 months		9.0 [7.9–10.3]	11. 8 [9.2–15.1]	NA	A–B: NS

Abbreviations: HTPs, heated tobacco products; NA, not applicable; NS, not significant (*p* > 0.05). * Rao-Scott Chi-Square tests accounted the complex survey design. Resulting test stats were design-based F for each pairwise comparison; † Values shown are median [interquartile range]. ‡ Although aggregated percentages were reported here, statistical tests were conducted using the five-point scales.

**Table 2:** Proportion of Four Subgroups of Concurrent Users.

Weighted % [95% Confidence Interval]		
	Daily HTP User (*n* = 550)	Non-daily HTP User (*n* = 258)
	48.8 [43.9–53.8]*	51.1 [46.2–56.0] *
Daily smoker (*n* = 3626)	Dual Daily User (*n* = 396)	Predominant Smoker (*n* = 213)
93.9 [91.2–95.9] †	48.4 [43.5–53.3]	45.5 [40.5–50.7]
Non-daily smoker (*n* = 212)	Predominant HTP User (*n* = 4)	Concurrent Non-daily User (*n* = 31)
6.1 [4.1–8.8] †	0.5 [0.2–1.3]	5.6 [3.7–8.3]

* Values shown are the sum of the overall column. † Values shown are the sum of the overall row.

**Table 3:** Comparison between Concurrent Daily Users and Concurrent Non-daily Users.

		(A) Concurrent Daily User (*n* = 609) *	(B) Concurrent Non-Daily User (*n* = 31)	Significance ^†^
		Weighted % [95% Confidence Interval]		
Sociodemographic				
Gender	Male	79.2 [74.0–83.7]	70.5 [47.6–86.3]	A-B: NS
	Female	20.7 [16.3–26.0]	29.5 [13.7–52.4]	
Age (years old)	20–29	18.1 [14.0–23.2]	40.3 [22.6–61.0]	A-B: 0.0146
	30–39	27.9 [23.6–32.6]	36.1 [19.2–57.4]	
	40–59	37.5 [32.9–42.4]	20.3 [7.4–44.8]	
	60 and older	16.5 [12.7–21.0]	3.2 [0.8–12.6]	
Annual Household Income	Low	15.8 [12.6–19.6]	22.4 [8.5–47.2]	A-B: NS
	Moderate	24.4 [20.2–29.1]	19.1 [8.5–37.5]	
	High	47.7 [42.6–52.8]	51.5 [31.6–71.0]	
	Refused/Do not know	12.1 [8.3–17.4]	7.0 [1.9–22.3]	
Education	Low	26.5 [22.6–30.7]	11.3 [4.4–26.3]	A-B: NS
	Moderate	21.2 [16.6–26.8]	19.0 [6.6–43.9]	
	High	52.1 [46.8–57.2]	67.5 [45.8–83.6]	
	Refused/Do not know	0.2 [0.1–0.9]	2.2 [0.3–14.4]	
Pattern of Product Use				
Cigarettes per day ‡		15.0 [10.0–20.0]	2.9 [1.3–6.0]	A-B < 0.0001
Tobacco-containing inserts per day ‡		6.0 [1.4–15.0]	1.4 [0.4–2.8]	A-B < 0.0001
Time to first tobacco product use	5 min or less	27.9 [23.8–32.5]	10.4 [3.1–29.7]	A-B < 0.0001
	6–30 min	45.6 [40.5–50.9]	18.2 [6.5–41.3]	
	31–60 min	16.0 [12.7–19.9]	27.3 [12.3–50.2]	
	More than 60 min	10.4 [7.8–13.8]	44.1 [25.6–64.4]	
Beliefs toward HTPs and cigarettes				
Believes HTPs are much or somewhat less addictive than cigarettes §		41.7 [36.8–46.8]	59.8 [38.7–77.9]	A-B: NS
Believes HTPs are much or somewhat less harmful to users than cigarettes§		68.9 [64.0–73.5]	84.1 [64.6–93.8]	A-B: NS
Believes secondhand emissions from HTP much or somewhat less harmful than secondhand emissions from cigarettes §		72.3 [67.3–76.7]	65.8 [43.0–83.1]	A-B: NS
Agrees society strongly or somewhat disapproves using HTPs §		23.6 [18.9–29.2]	13.2 [4.3–34.2]	A-B: NS
Agrees society strongly or somewhat disapproves smoking cigarettes §		65.3 [60.5–69.8]	46.5 [27.4–66.6]	A-B: NS
Has very positive or positive overall opinions of HTPs §		55.9 [50.6–61.0]	58.5 [37.1–77.1]	A-B: NS
Has very positive or positive overall opinions of cigarettes §		46.3 [41.2–51.5]	39.2 [21.7–60.0]	A-B: NS
Smoking Cessation-related Behaviors				
Attempted to quit at least once in the last 12 months		51.9 [44.7–58.9]	89.4 [67.0–97.2]	A-B: 0.0013
Plans to quit smoking cigarettes in the next 6 months		9.3 [7.0–12.3]	50.6 [30.2–70.9]	A-B < 0.0001

Abbreviations: HTPs, heated tobacco products; NS, not significant (*p* > 0.05). * Comprised of dual daily user (n = 434) and predominant smokers (n = 219). † Rao-Scott Chi-Square tests accounted the complex survey design. Resulting test stats were design-based F for each comparison. ‡ Values shown are median [interquartile range]. § Although aggregated percentages were reported here, statistical tests were conducted using the five-point scales.

**Table 4:** Comparison of four groups of daily users.

		(A) Exclusive Daily Smoker (*n* = 3017)	Concurrent Daily Use *		(D) Exclusive Daily HTP User (*n* = 150)	Significance †
			(B) Predominant Smoker (*n* = 213)	(C) Dual Daily User (*n* = 396)		
		Weighted %				
Sociodemographic						
Gender	Male	68.7	80.1	78.5	71.6	A-B: 0.0369
	Female	31.3	19.9	21.5	28.4	B-C: NS
						C-D: NS
Age	20–29	8.5	12.2	23.7	11.1	A-B: NS
	30–39	18.7	26.6	29.1	22.9	B-C: 0.0036
	40–59	41.7	37.3	37.7	58.0	C-D: 0.0070
	60 and older	31.0	24.0	9.4	7.9	
Annual Household Income	Low	28.6	14.9	16.7	15.8	A-B: 0.0202
	Moderate	22.2	24.4	24.3	18.8	B-C: NS
	High	35.4	46.3	49.0	59.7	C-D: NS
	Refused/Do not know	13.9	14.4	10.0	5.6	
Education	Low	32.8	23.5	29.2	23.1	A-B: NS
	Moderate	22.1	22.4	20.1	26.2	B-C: NS
	High	43.9	54.1	50.2	50.2	C-D: NS
	Refused/Do not know	1.2	-	0.5	0.5	
Pattern of Product Use						
Cigarettes per day‡		15.0 (10.0–20.0)	18.0 (10.0–20.0)	15.0 (10.0–20.0)	NA	A-B: 0.0309
						A-C: NS
Tobacco-containing inserts per day‡		NA	0.7 (0.3–1.4)	10.0 (5.0–15.0)	10.0 (7.0–20.0)	B-D<0.0001
						C-D: 0.0076
Time to first tobacco product use	5 min or less	27.5	27.2	28.6	20.1	A-B: NS
	6–30 min	40.9	46.2	45.1	40.7	B-C: NS
	31–60 min	16.0	15.0	16.9	20.3	C-D: NS
	More than 60 min	15.6	11.6	9.4	18.8	
Beliefs toward HTPs and cigarettes						
Believes HTPs are much or somewhat less addictive than cigarettes§		20.0	38.9	44.3	47.7	A-B < 0.0001
						B-C: NS
						C-D: NS
Believes HTPs are much or somewhat less harmful to users than cigarettes§		44.2	64.8	72.8	90.0	A-B<0.0001
						B-C: NS
						C-D: 0.0001
Believes secondhand emissions from HTP much or somewhat less harmful than secondhand emissions from cigarettes§		50.7	70.3	74.2	87.3	A-B<0.0001
						B-C: NS
						C-D<0.0001
Agrees society strongly or somewhat disapproves using HTPs§		23.4	27.5	20.0	28.8	A-B: NS
						B-C: 0.0307
						C-D: NS
Agrees society strongly or somewhat disapproves smoking cigarettes§		64.9	70.9	60.0	62.6	A-B: NS
						B-D: NS
						C-D: NS
Has very positive or positive overall opinions of HTPs§		28.2	47.9	63.4	64.7	A-B < 0.0001
						B-C: 0.0160
						C-D: NS
Has very positive or positive overall opinions of cigarettes§		37.9	49.1	43.7	37.7	A-B: 0.0257
						B-C: NS
						C-D: NS
Smoking Cessation-related Behaviors						
Attempted to quit at least once in the last 12 months		49.3	48.8	54.3	NA	A-B: NS
						A-C: NS
Plans to quit smoking cigarettes in the next 6 months		8.0	4.9	14.0	NA	A-B: NS
						A-C: 0.0017

Abbreviations: HTPs, heated tobacco products; NA, not applicable; NS, not significant (*p* > 0.05). * Predominant HTP users were excluded in this table due to small number of samples (*n* = 5). † Rao-Scott Chi-Square tests accounted the complex survey design. Resulting test stats were design-based F for each pairwise comparison. ‡ Values shown are median [interquartile range]. § Although aggregated percentages were reported here, statistical tests were conducted using the five-point scales.

**Table 5:** Comparison of three groups of non-daily users.

		(A) Exclusive Non-Daily Smoker (*n* = 177)	(B) Concurrent Non-Daily User (*n* = 31)	(C) Exclusive Non-Daily HTP User (*n* = 14)	Significance *
		Weighted %			
Sociodemographic					
Gender	Male	78.8	70.5	72.6	A-B: NS
	Female	21.2	29.5	27.4	B-C: NS
Age	20–29	24.9	40.3	21.6	A-B: NS
	30–39	26.8	36.1	25.6	B-C: NS
	40–59	32.3	20.3	49.0	
	60 and older	16.0	3.2	3.7	
Annual Household Income	Low	34.8	22.4	-	A-B: NS
	Moderate	17.4	19.1	29.6	B-C: NS
	High	36.8	51.5	62.5	
	Refused/Do not know	11.0	7.0	7.9	
Education	Low	23.5	11.3	35.9	A-B: NS
	Moderate	21.1	19.0	7.9	B-C: NS
	High	54.1	67.5	56.2	
	Refused/Do not know	1.3	2.2	-	
Pattern of Product Use					
Cigarettes per day †		1.4 (0.7–2.9)	2.9 (1.3–6.0)	NA	A-B: 0.0017
Tobacco-containing inserts per day†		NA	1.4 (0.4–2.8)	1.7 (0.7–7.1)	B-C: NS
Time to first tobacco product use	5 min or less	3.2	10.4	11.6	A-B: 0.0200
	6–30 min	13.6	18.2	22.6	B-C: NS
	31–60 min	9.5	27.3	14.7	
	More than 60 min	73.7	44.1	51.1	
Beliefs toward HTPs and cigarettes					
Believes HTPs are much or somewhat less addictive than cigarettes ‡		20.1	59.8	49.9	A-B: 0.0002
					B-C: NS
Believes HTPs are much or somewhat less harmful than cigarettes ‡		35.9	84.1	73.5	A-B: 0.0008
					B-C: NS
Believes secondhand emissions from HTP much or somewhat less harmful than secondhand emissions from cigarettes ‡		43.4	65.8	78.1	A-B: NS
					B-C: NS
Agrees society strongly or somewhat disapproves using HTPs ‡		23.8	13.2	35.3	A-B: 0.0422
					B-C: NS
Agrees society strongly or somewhat disapproves smoking cigarettes ‡		63.9	46.5	36.4	A-B: NS
					B-C: 0.0317
Has very positive or positive overall opinions of HTPs ‡		24.5	58.5	44.2	A-B: 0.0202
					B-C: NS
Has very positive or positive overall opinions of cigarettes ‡		28.6	39.2	7.5	A-B: NS
					B-C: NS
Smoking Cessation-related Behaviors					
Attempted to quit at least once in the last 12 months		68.3	89.4	NA	A-B: NS
			
Plans to quit smoking cigarettes in the next 6 months		25.9	50.6	NA	A-B: 0.0215
			

Abbreviations: HTPs, heated tobacco products; NA, not applicable; NS, not significant (*p* > 0.05). * Rao-Scott Chi-Square tests accounted the complex survey design. Resulting test stats were design-based F for each pairwise comparison. † Values shown are median [interquartile range]. ‡ Although aggregated percentages were reported here, the statistical tests were conducted using the five-point scales
